# Different Types of ANCA Determine Different Clinical Phenotypes and Outcome in ANCA-Associated Vasculitis (AAV)

**DOI:** 10.3389/fmed.2021.783757

**Published:** 2022-01-21

**Authors:** Kostas Bantis, Maria J. Stangou, Savvas Kalpakidis, Christina Nikolaidou, George Lioulios, Zoi Mitsoglou, Fotini Iatridi, Asimina Fylaktou, Aikaterini Papagianni

**Affiliations:** ^1^Department of Nephrology, Hippokration Hospital, Aristotle University of Thessaloniki, Thessaloniki, Greece; ^2^Department of Pathology, Hippokration Hospital, Thessaloniki, Greece; ^3^Department of Immunology, National Peripheral Histocompatibility Center, Hippokration Hospital, Thessaloniki, Greece

**Keywords:** ANCA–associated vasculitis, crescentic, sclerotic, outcome, relapse, clinical phenotype

## Abstract

**Aim:**

Accumulating evidence supports the use of antineutrophil cytoplasmic antibody (ANCA) type to classify different clinical entities. We aimed to evaluate whether the presence and type of ANCA determine different diseases, based on clinical phenotypes, renal involvement, and response to treatment.

**Patients and Methods:**

Differences in terms of clinical manifestations, disease activity, laboratory parameters, and histology were recorded between patients with focal necrotizing glomerulonephritis (FNGN) due to myeloperoxidase (MPO-), proteinase 3-ANCA(+) [PR3-ANCA(+)], and ANCA(-) disease at time of diagnosis. Patients were treated with the same protocol and followed-up for 24 months, in a scheduled basis of every month for the first year and every 3 months for the second year. Primary end points were: (i) Combined end-stage renal disease (ESRD) and/or death and (ii) The presence of major or minor relapse during follow-up and secondary endpoint was the combination of ESRD and reduction of estimated glomerular filtration rate (eGFR) ≥ 50%.

**Results:**

A total of 92 patients (M/F 39/53, mean age 59.1 ± 15 years) diagnosed with FNGN due to ANCA-associated vasculitis (AAV), 36 (39.1%) patients diagnosed with PR3-ANCA, 39 (42.4%) patients diagnosed with MPO-ANCA, and 17 (18.5%) patients diagnosed with ANCA(-) were included. Number of involved systems differed significantly between PR3-, MPO-ANCA, and ANCA(-), with only renal involvement in 3, 25.5, and 29% of patients, two systems involved in 33, 31, and 59% of patients, and > 3 systems involved in 64, 43.5, and 12% of patients, respectively (*p* = 0.002). Histology classification revealed focal, crescentic, mixed, and sclerotic type in 14, 64, 19, and 3% of PR3-ANCA(+), 8, 28, 18, and 46% of MPO-ANCA, and 41, 29, 6, and 24% of ANCA(-), respectively (*p* < 0.0001). Primary end point of ESRD ± Death was reached in 11 (30.6%), 16 (41%), and 6 (35.5%) patients with PR3-ANCA(+), MPO-ANCA(+), and ANCA(-), respectively (*p* = NS); similarly, ESRD± > 50% eGFR reduction in 8 (22.2%), 15 (38.5%), and 5 (29.4%) patients, respectively (*p* = NS), meaning that patients with MPO-ANCA(+) showed a propensity to decline renal function. Rate of relapse was increased in the presence of patients with PR3-ANCA(+), 14 (38.9%), 4 (11.8%), and 2 (10.3%) of patients with PR3-ANCA(+), MPO-ANCA(+), and ANCA(-), had at least one relapse during the two-year follow-up (*p* = 0.006).

**Conclusion:**

Clinical phenotype and renal histology differ significantly between PR3-ANCA(+), MPO-ANCA(+), and ANCA(-) disease and FNGN; however, renal function outcome is similar, despite the increased rate of relapses in patients with PR3-ANCA(+).

## Introduction

Antineutrophil cytoplasmic antibodies (ANCAs)-associated vasculitis (AAV) is a group of autoimmune disorders affecting small and medium size vessels, characterized by necrotizing inflammatory reactions and their clinical consequences extent to a great spectrum of systems and organs including respiratory system, kidneys, skin, central and peripheral nervous system, gastrointestinal system, etc. Kidneys are affected at a high rate of 60–80% of cases, causing focal necrotizing glomerulonephritis (FNGN) and rapidly deteriorating kidney function in most cases, which may lead to end-stage renal disease (ESRD), if diagnosis and appropriate treatment are delayed (1).

The disease is characterized by the presence of antibodies directed mainly against proteinic targets located in the cytoplasmic granules of neutrophils. Recent studies have proved the pathogenic significance of ANCAs, during all the stages of disease progression, as they cause neutrophil stimulation, complement activation, aggregation of lymphocytes, macrophages, and platelets, which infiltrate vessel walls, causing necrotizing inflammation, destruction of small- and medium-sized vessels leading to necrosis of the tissues supplied (2, 3). Traditionally, AAVs include four clinical syndromes, namely, microscopic polyangiitis (MPA), granulomatosis with polyangiitis (GPA), eosinophilic GPA (EGPA), and renal limited disease (RLD) (4). Clinical syndromes have been described in an attempt to define distinct clinical entities in the spectrum of AAVs and distinguish patients in separate groups to better understand and approach pathogenesis and treatment. However, additional problems have raised; since, the description of clinical syndromes, substantial overlap between myeloperoxidase (MPO), GPA, and RLD do occur, in terms of type and clinical symptomatology of ANCA. Furthermore, the pathogenetic significance of ANCA, along with their importance in determining clinical presentation, disease activity, response to treatment, and predicting relapse, supports their use as biomarkers of the disease, but also forces investigators to rely on the presence and type of ANCA in order to discriminate different clinical phenotypes.

In this study, we evaluated the differences between MPO-ANCA(+), [proteinase 3 (PR3)-ANCA(+)], and ANCA(-) vasculitis in terms of clinical symptoms at presentation and severity of renal pathology; in addition, we prospectively assessed differences in response to treatment, the outcome of the disease, and rate of relapse in patients with necrotizing glomerulonephritis (NGN) due to AAV (5, 6).

## Patients and Methods

### Patients

This prospective, longitudinal, and observational study included adult patients with NGN due to ANCA(+) vasculitis. Diagnosis in all the patients was based on renal biopsy findings, optical microscopy, and immunostaining (immunofluorescence and/or immunohistochemistry), showing pauci-immune NGN. Initial presentation of patients consisted of renal and/or extrarenal manifestations of ANCA(+) vasculitis; however, they were referred to our department, if they showed micro- or macroscopic hematuria, proteinuria, and/or renal function impairment.

Inclusion criteria were: age > 18 years old, renal biopsy at the time of presentation or not later than 1 month after presentation, induction, and maintenance treatment with the standard protocol, based on the Kidney Disease: Improving Global Outcomes (KDIGO) guidelines. Only patients with newly diagnosed AAV were included and those patients with the known disease who presented with a relapse episode were excluded. Patients with evidence of systemic lupus erythematosus, rheumatoid arthritis, immunoglobulin A (IgA) vasculitis, cryoglobulinemia, abnormal immunoglobulin levels, antinuclear antibodies (ANAs), positive anti-glomerular basement membrane (GBM) antibodies or double-positive ANCA, and anti-GBM were excluded from this study. In addition, patients who had a recent or chronic infection and/or recent (<12 months) treatment with steroids or immunosuppressants were also excluded. All the patients signed the informed consent before participating. The study was performed in line with the principles of the Declaration of Helsinki. Approval was granted by the Ethics Committee of the Hippokration General Hospital, Thessaloniki, Greece, Approval Number 12/16.

### Histology Assessment

All the renal biopsies were undergone assessment on both optical microscopies, using the routine stains including H&E, periodic acid–Schiff (PAS), periodic acid silver methenamine (PASM), and Masson's trichrome and immunostaining (immunofluorescence and/or immunohistochemistry) including immunoglobulins (IgA, IgG, and IgM), complement components (C3, C1q, and C4), fibrin, kappa, and lambda light chains.

The percentage of crescents, including cellular, fibrocellular, and fibrous crescents, was estimated on optical microscopy as well as the degree of tubule-interstitial infiltration, interstitial fibrosis, and tubular atrophy. The degree of tubule-interstitial infiltration was semiquantively assessed, based on a scale of 0–2 (where 0 was substituted for absence or mild, 1 for moderate, and 2 for severe). Assessment of the severity of tubular atrophy and interstitial fibrosis was based on the percentage of tubules with characteristic findings of atrophy and estimated on a scale of 0–3 (where 0 was substituted for no abnormalities, 1 for < 30%, 2 for 31–60%, and 3 for > 60% of tubules with atrophy).

Based on the criteria established by the Immunonephrology Working Group (IWGRP), all the biopsies were categorized into four classes: focal (≥50% normal glomeruli); crescentic (≥50% glomeruli with cellular crescents); mixed (<50% normal, <50% crescentic, and <50% globally sclerotic glomeruli); and sclerotic (≥50% globally sclerotic glomeruli).

### Treatment Protocol

Treatment was based on the KDIGO guidelines; the same therapeutic protocol was applied to all the patients, including induction treatment with intravenous pulses of 500 mg prednisolone, given daily for 6 consecutive days, followed by oral prednisolone 1 mg/kg/day and intravenous (IV) cyclophosphamide at doses adjusted to age and renal function of the patient. Prednisolone doses were gradually reduced by 5 mg, every 2 weeks. Cyclophosphamide was administered at 10 pulses, followed by azathioprine or mycophenolate mofetil.

Plasmapheresis was performed, according to the KDIGO guidelines, in case of severe renal failure or pulmonary hemorrhage. Seven to ten plasma exchanges of 3,000 ml were performed in a day-by-day basis.

A kidney biopsy was performed either just before initiation of therapy or within 1 month, in cases with increased risk of bleeding or those requiring acute treatment.

### Patients Follow-Up

Day of study entry was the day of starting treatment. Clinical parameters including extrarenal manifestations, namely, pulmonary, myoskeletal, central, and peripheral nervous system involvement, the Birmingham Vasculitis Activity Score (BVAS) and the Five-Factor Score-2009 (FFS-2009), laboratory investigation, including renal function, degree of proteinuria and serum levels of ANCA, and histological findings were estimated.

Patients were regularly followed-up on an outpatients basis, every month for the first year and every 3 months for the second year. In a case of a sudden event, such as relapse, infection, deterioration of renal function, or an acute event, patients were followed according to decision of the physician.

Parameters recorded at time of diagnosis were estimated glomerular filtration rate (eGFR), the BVAS and the FFS-2009 score, white cell count (WCC), neutrophil, lymphocyte, and platelets count, neutrophil/lymphocyte ratio (NLR) and platelet/lymphocyte ratio (PLR), and the need of hemodialysis (HD). During follow-up, eGFR was assessed every month for the first year and every 3 months for the second year and recorded at 3 (eGFRm3), 6 (eGFRm6), 12 (eGFRm12), and at the end of 24 months (eGFRm24). The slope of eGFR was estimated based on serial measurements of eGFR.

Primary endpoints were: (i) Combined event of ESRD and/or death and (ii) The presence of major or minor relapse of AAV during follow-up. As a secondary endpoint, the combination of ESRD and reduction of eGFR by ≥ 50% was defined. Patients were followed for 24 months; for those patients who reached ESRD and started on HD or patients who died, follow-up period lasted until the day of starting on HD or the day of death.

### Definitions

Estimated glomerular filtration rate was estimated by the formula Chronic Kidney Disease Epidemiology Collaboration (CKD-EPI).

The BVAS version 3 score was calculated for all the patients. A total of 56 items, representing involvement of different systems or organs, including upper and lower respiratory, cardiovascular, abdominal, renal, and nervous system manifestations, cutaneous, mucous, and general indices, were organized into nine different groups.

The FFS-2009 score involved five factors and five points, such as age, renal insufficiency, cardiac involvement, gastrointestinal, and upper respiratory [ear-nose-throat (ENT)] manifestations. ENT signs were assessed in all the patients, regardless the presence or type of ANCA. The absence of ENT manifestations in presence of the rest parameters was considered as +1 point.

Major relapse was defined as life- or organ-threatening relapse after achieving complete or partial remission. Minor relapse was defined as an increase in disease activity after complete or partial remission, but not causing life- or organ-threatening complications.

### Statistics

The Statistical Package for the Social Sciences (SPSS) (SPSS Incorporation, Chicago, Illinois, USA) version 25.0 for windows was used for the statistical analysis. Normality of the distribution for continuous variables was estimated by the Kolmogorov–Smirnov and the Shapiro–Wilk tests. Values of *p* < 0.05 (two-tailed) were considered statistically significant for all the comparisons.

Mean ± SD or medians and interquartile range (IQR) were used to describe data in normally distributed and nonparametric variables, respectively. In the same way, the Student's *t*-test for nonpaired or the ANOVA test and the Mann–Whitney *U* test or the Wilcoxon signed-rank test, respectively, were performed to compare differences between groups.

## Results

### Characteristics of Patients at Time of Diagnosis

We included 92 patients, M/F 39/53, mean age 59.1 ± 15 years, who were diagnosed with FNGN due to AAV in the period 1/2016–1/2019. A total of 39 patients (42.4%) had MPO-ANCA, 36 (39.1%) patients had PR3-ANCA, and 17 (18.5%) patients were ANCA negative.

At the time of diagnosis, median levels of proteinuria Urine protein (Upr) were 1.49 (0.2–11) g/24 h and eGFR was 13.8 (6.15–98.2) mg/dl/1.73 m^2^. A total of 32 patients (34.8%) were dialysis-dependent at presentation and eGFR in the rest 60 patients was 22 (10.8–98.2) mg/dl/1.73 m^2^.

### Differences According to the Type of ANCA

Clinical characteristics, manifestations of vasculitis, disease activity and laboratory findings, and differences between ANCA types are shown in Tables 1, 2.

**Table 1:** Differences in the clinical symptoms and extrarenal manifestations between patients with myeloperoxidase-antineutrophil cytoplasmic antibody (MPO-ANCA)(+), proteinase 3 (PR3)-ANCA(+), and ANCA(-).

	**MPO-ANCA (+)**	**PR3-ANCA (+)**	**ANCA (-)**	***p***
N (%)	39 (42.4)	36 (39.2)	17 (18.4)	
Females	24 (61.5)	21 (58.3)	8 (47.1)	NS
Extrarenal manifestations				
Fever	17 (43.6)	12 (33.3)	2 (11.8)	0.05
Skin rash	8 (20.5)	8 (22.2)	3 (17.6)	NS
ENT	4 (10.3)	12 (33.3)	2 (11.8)	0.03
Lower respiratory system	16 (41)	26 (72.2)	6 (35.3)	0.007
CNS involvement	0	2 (5.6)	0	NS
Arthritis	9 (23.1)	18 (50)	4 (23.5)	0.03
Gastroinenstinal involvement	0	1 (2.8)	0	NS
HD dependent	20 (51.3)	8 (22.2)	4 (23.5)	0.01
BVAS score	15 (10–24)	17 (10–25)	14 (10–25)	NS
FFS score	2 (0–3)	2 (0-3)	2 (0–3)	NS

**Table 2:** Laboratory findings in patients with MPO-ANCA(+), PR3-ANCA(+), and ANCA(-) at the time of presentation.

	**MPO-ANCA(+)**	**PR3-ANCA(+)**	**ANCA (-)**	***p***
Age (yrs)	60.6, 14.9	59.4, 12.4	55.2, 19	NS
WCC (K/μl)	9701, 4260	10785, 4024	10207, 2456	NS
Neutrophils (%)	81, 8.8	77, 11.8	77, 12.9	NS
Neutrophils	8042, 3954	8604, 4097	7965, 2581	NS
Lymphocytes (%)	11.5, 6.9	15.1, 8.7	15.8, 10.5	NS
Lymphocytes (K/μl)	952, 612	1356, 683	1503, 842	0.02
NLR	11.5, 8.5	7.7, 4.5	9.1, 8.3	NS
PLT (K/μl)*10^3^	255, 96	359, 100	309, 134	0.01
PLR	344, 191	309, 141	391, 543	NS
Hb (g/dl)	10, 1.4	11.1, 1.5	10.09, 1.6	NS
Ht (%)	30.6, 4.5	33.5, 4.5	30.5, 5.1	NS
e-GFR (mL/min/1.73 m^2^)	20.5, 12.9	22.4, 15.8	21.4, 15.2	NS
Uprot (g/24 h)	1.6, 1.1	1.7, 1.6	3.5, 3.3	NS

### Extrarenal Manifestations

**Figure 1:**
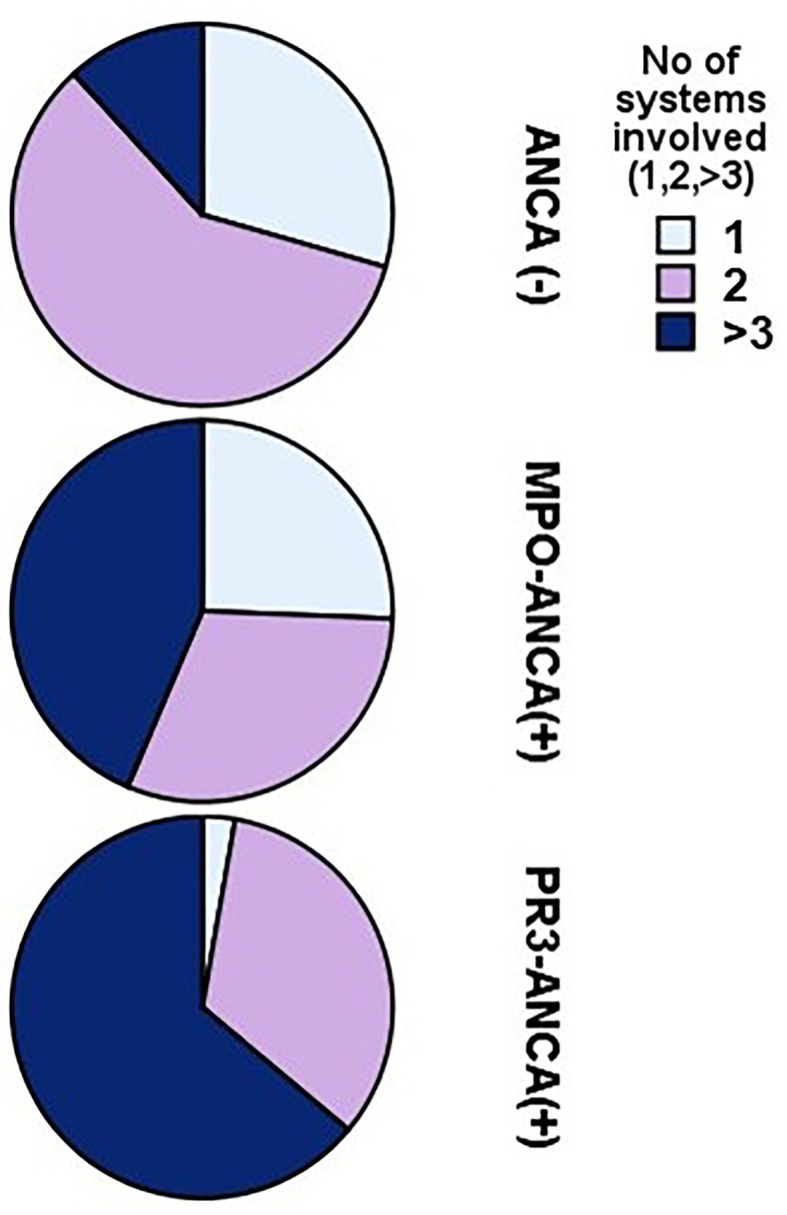
Differences in the frequency of organ systems involvement according to the type of antineutrophil cytoplasmic antibody (ANCA).

The number of extrarenal manifestations was estimated for patients with MPO-ANCA(+), PR3-ANCA(+), and ANCA(-) and results are given in Figure 1 and are shown in detail in Table 3.

**Table 3:** Numbers and percentage of patients presenting with 1, 2, or > 3 systems involved.

No of Exrarenal Manifestations^*^	**ANCA (-)**	**PR3- ANCA**	**MPO-ANCA**
1	5 (29)	1 (3)	10 (25.5)
2	10 (59)	12 (33)	12 (31)
>3	2 (12)	23 (64)	17 (43.5)

* *Pearson chi-square test 17, p = 0.002, likelihood ratio 20.2, p < 0.0001*.

The presence of PR3-ANCA was followed by advanced extrarenal organ involvement. In patients with PR3-ANCA(+), only 3% presented with clinical symptoms restricted to kidneys, while 64% had more than three systems involved, compared to patients with 43.5% in MPO-ANCA(+) and only 12% in ANCA(-).

### Renal Biopsy Findings

From the whole cohort of patients, 74 (80.4%) patients had focal necrosis on renal biopsy and 14 (15.2%) patients had endocapillary hyperplasia. A median number of glomeruli with crescent formation was estimated to be 56.25 (0–100), 23 (0–100) of them were cellular, 12.38 (0–100) were fibrocellular, and 8.37 (0–50) were fibrous crescents. Severity of interstitial fibrosis was estimated as 0 in 11 (12%), 1 in 50 (54.3%), and 2 in 31 (33.7%) patients; inflammatory infiltration of interstitium was graded as 0 in 6 (6.5%), 1 in 45 (49%), and 2 in 41(44.5%) patients.

**Figure 2:**
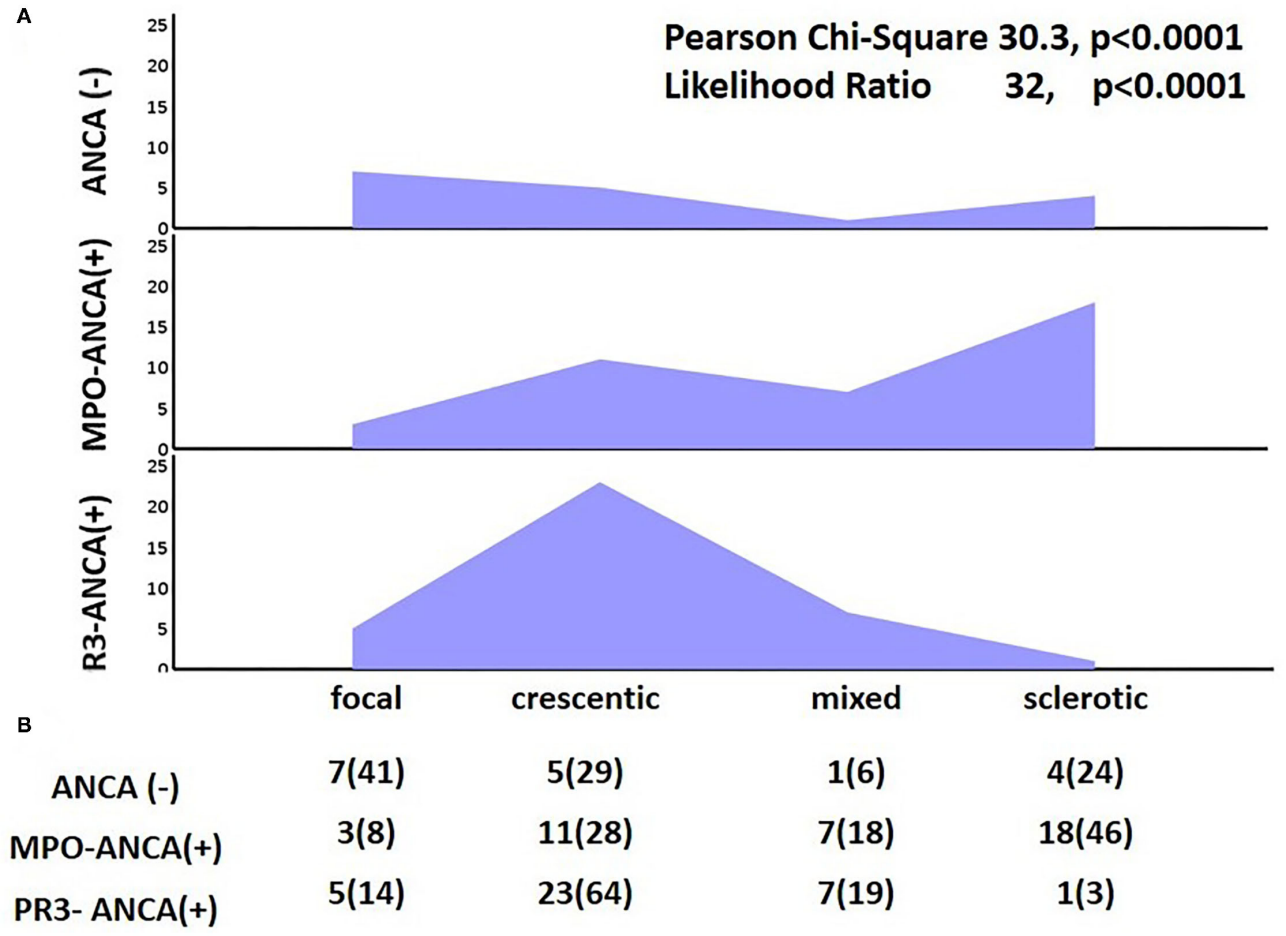
Histological classification of renal biopsy findings; **(A)** depicts the ratio of focal, crescentic, mixed, and sclerotic changes in patients with ANCA(-), myeloperoxidase (MPO)-ANCA(+), and proteinase 3 (PR3)-ANCA(+) and **(B)** describes number and percentage of patients with the above changes.

When histological findings were classified into four different classes (focal, crescentic, mixed, and sclerotic) according to the IWGRP (7), we noticed a significant difference between patients with ANCA(-), MPO-ANCA(+), and PR3-ANCA(+), as shown in Figure 2. The crescentic type had a significantly increased incidence in patients with PR3-ANCA(+), almost 64% of them presented with the crescentic type compared to <30% in both the patients with MPO-ANCA(+) and ANCA(-). Instead, the sclerotic type had a privilege in patients with MPO-ANCA(+), 46% of whom had a sclerotic type, compared to patients with 24% of ANCA(-) and only 3% of PR3-ANCA(+).

### Renal Function Outcome

**Figure 3:**
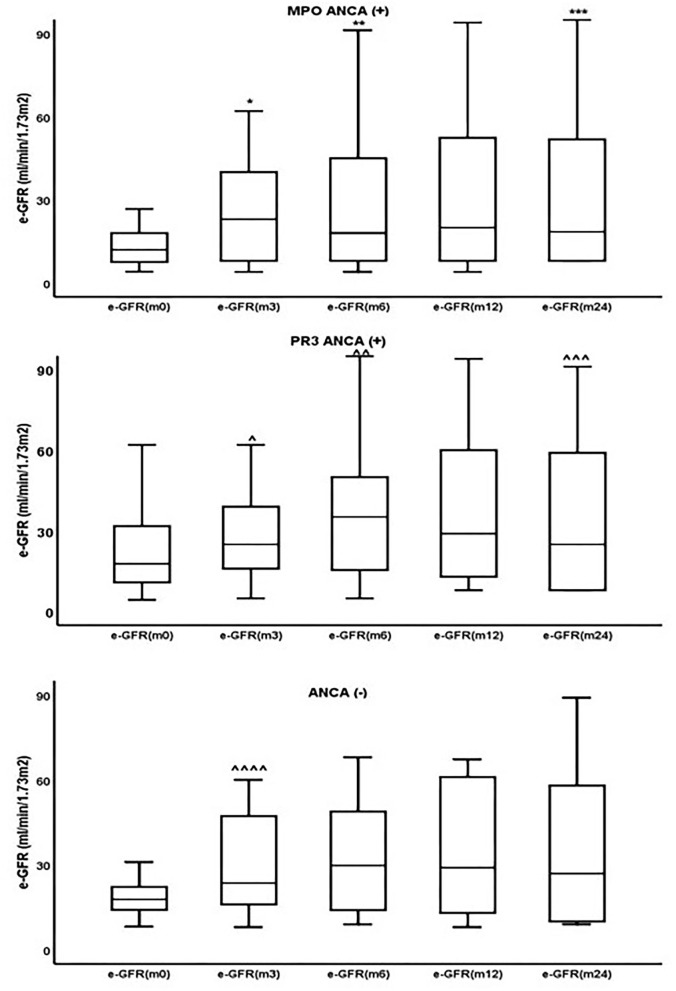
Changes in estimated glomerular filtration rate (eGFR) during follow-up in the three groups of patients. *p [eGFR (m3) vs. eGFR (m0)] = 0.004, **p [eGFR (m6) vs. eGFR (m3)] = 0.01, ***p [eGFR (m24) vs. eGFR (m12)] = 0.004, ^∧^p [eGFR (m3) vs. eGFR (m0)] = 0.001, ^∧∧^p [eGFR (m6) vs. eGFR (m3)] = 0.04, ^∧∧∧^p [eGFR (m24) vs. eGFR (m12)] <0.0001, ^∧∧∧∧^p [eGFR (m3) vs. eGFR (m0)] = 0.05.

Changes in eGFR for the whole cohort of patients and separately for MPO-ANCA(+), PR3-ANCA(+), and ANCA(-) are shown in Table 4 and Figure 3.

**Table 4:** Renal function outcome in the three groups of patients such as MPO-ANCA(+), PR3-ANCA (+), and ANCA(-).

	**e-GFR (m0)**	**e-GFR (m3)**	**e-GFR (m6)**	**e-GFR (m12)**	**e-GFR (m24)**	***p***
MPO(+) ANCA	20.5 ± 22.9	28.5 ± 24.2	32.1 ± 26.8	32.9 ± 29.4	31.7 ± 28.7	0.001
PR3(+) ANCA	22.4 ± 15.8	34.7 ± 22	38.2 ± 24.9	38.7 ± 25.8	36.5 ± 26.2	0.003
ANCA (-)	21.4 ± 15.2	28.2 ± 17.8	31.3 ± 21.6	32.2 ± 22.5	34.5 ± 25.8	0.05

Although there were differences in renal function at the time of diagnosis and during follow-up between patients with MPO-ANCA(+), PR3-ANCA(+), and ANCA(-), these differences could not reach statistical significance.

The primary endpoint of ESRD ± Death was reached in 16 (41%), 11 (30.6%), and 6 (35.5%) patients with MPO-ANCA(+), PR3-ANCA(+), and ANCA(-), respectively (*p* = NS); similarly, the secondary endpoint of ESRD± > 50% reduction in eGFR was reached by 15 (38.5%), 8 (22.2%), and 5 (29.4%) patients, respectively (*p* = NS), meaning that there was a tendency of patients with MPO-ANCA(+) to decline renal function, though not reaching statistical significance. However, with respect to the relapse rate, there was a clear preponderance of patients with PR3-ANCA(+). The percentage of patients who had at least one, major or minor, relapse during the 2-year follow-up was 14 (38.9%), 4 (11.8%), and 2 (10.3%) in patients with PR3-ANCA(+), MPO-ANCA(+), and ANCA(-) (*p* = 0.006).

### Impact of Histology in the Outcome of Renal Function

Outcome of renal function was significantly associated with histological classification. Thus, primary end point was reached in 3/15 (20%), 10/39 (25.6%), 3/15 (20%), and 17/23 (73.9%) patients with focal, crescentic, mixed, and sclerotic type on renal biopsy, respectively (*p* < 0.0001). Similarly, secondary end point was reached in 2/15 (13.3%), 8/39 (20.5%), 2/15 (13.3%), and 17/23 (73.9%) patients, respectively (*p* < 0.0001).

However, there were no significant differences when we compared outcomes of patients in the same histological type according to ANCA type, probably because of the relatively small number of patients.

## Discussion

During the last few years, there is accumulated evidence, which supports the use of ANCA specificity rather than the use of clinical syndromes, MPA, GPA, EGPA, and RLD, to categorize the AAVs (6, 8). We performed a prospective study in patients with renal involvement, in particular FNGN, due to AAV, to investigate whether specific clinical presentation, histology, and outcome of MPO-ANCA(+), PR3-ANCA(+), and ANCA(-) diseases support their definition as different entities.

In this study, 92 patients with biopsy-proven NGN due to AAV were included. Thirty nine of them, 42% were MPO-ANCA(+), 36 (39%) were PR3-ANCA(+), and 17 (19%) were ANCA(-). Epidemiologically, AAV is a rare autoimmune disease with a high mortality risk, if diagnosis is delayed. The incidence of the disease is estimated to be 13–30 cases per million population in North America and Europe (9, 10). Geographically, there seems to be a difference in the incidence of the two types of AVV, with PR3-ANCA(+) disease being more prevalent and northern parts of the world and MPO-ANCA(+) predominating in the south, southern Europe, and Asia (11). In agreement with these finding, this study, which included Caucasian population, mainly from Southern Europe, proved a slightly higher prevalence of MPO-ANCA, compared to PR3-ANCA and ANCA(-).

Patients with PR3-ANCA showed an increased frequency of extrarenal manifestations at presentation including involvement of upper and lower respiratory system and arthritis. On the other hand, MPO-ANCA-associated vasculitis often presented with advanced renal impairment, with more than 50% of patients with MPO-ANCA(+) requiring hemodialysis at time of diagnosis, compared to only 22 and 23.5% of patients with PR3-ANCA(+) and ANCA(-), respectively.

Our findings support recent evidence that the two types of ANCA-associated vasculitis may represent two distinct diseases, accompanied by rather fewer common and more divergent characteristics. Indeed, in this study, MPO-ANCA(+) vasculitis, apart from the impaired renal function, seemed to be placed more closely to ANCA(-) vasculitis rather than PR3-ANCA(+), in terms of the rest clinical and laboratory characteristics.

The two diseases have differences not only in the frequency of specific organ involvement, but also in the way precise organs are affected. For instance, the upper respiratory system is more frequently affected in PR3-ANCA(+) and kidneys predominated in MPO-ANCA(+) vasculitis (12, 13). Moreover, patients with MPO-ANCAs experience more severe renal disease at presentation, which is due to the increased frequency of chronic lesions in renal biopsy (13). Correspondingly, they express more severe pulmonary interstitial fibrosis, whereas lung involvement in PR3-ANCA(+) disease usually presents with cavitary lesions or nodules. Alveolar hemorrhages equally found in both the ANCA types, although other manifestations, such as destructive nose, ear, and throat, seem to be more common in patients with PR3-ANCA(+) (5, 14). In our patients, we described significant differences in the renal histology, crescentic type was prominent in PR3-ANCA(+), while the sclerotic type of AVV was the predominant type among patients with MPO-ANCA(+).

Different clinical phenotypes and different histology phenotypes may represent diversity in disease pathogenesis, depending on the presence and type of ANCA. *In-vivo* studies have proved the pathogenic significance of both the MPO- and PR3-ANCA through the initiation and maintenance of inflammatory reactions (15–18). Even more importantly, the genetic background of AVV has also been proved, with studies confirming the association of AVVs with the major histocompatibility complex (MHC) and specifically with gene variants in HLA-DPA1 and DPB1 for PR3-ANCA-AAV and HLA-DQA2 and DQB1 for MPO-ANCA-AAV (19, 20). The above studies confirmed that MPA and GPA are genetically distinct autoimmune diseases, although the genetic profile seems to be much stronger associated with ANCA specificity rather than the clinical phenotype. Beyond the genetic basis, further issues are still arising, in the use of clinical AAV syndromes, namely, MPA, GPA, and EGPA. There is a clear overlap between them, as they share common clinical features and their classification is rather subjective and based to initial disease features (5, 19, 20).

There is a decent percentage of patients, around 30%, that present with clinicopathological features of AAV, but their ANCA titles are negative. A variety of reasons may be implicated including differences in ANCA detection methods, the timing of testing, or the presence of antigens not yet determined (21). Patients with ANCA(-) often have a renal limited disease rather than generalized multisystemic disease, lesser extrarenal manifestations, and are of younger age compared to ANCA(+) (22). However, to the best of our knowledge, for this group of patients is limited, since it is a group that is often excluded from the studies and, thus, more studies need to be conducted to reach assertive results and information.

Although there were differences in renal function at the time of diagnosis and during follow-up between patients with MPO-ANCA(+), PR3-ANCA(+), and ANCA(-) and there also was a significant difference in the need for dialysis in the group of patients with MPO-ANCA and these differences could not reach statistical significance. Our primary endpoint of either ESRD and/or death was reached in 16 (41%), 11 (30.6%), and 6 (35.5%) patients with MPO-ANCA(+), PR3-ANCA(+), and ANCA(-), respectively, and the secondary endpoint of ESRD± > 50% reduction in eGFR in 15 (38.5%), 8 (22.2%), and 5 (29.4%) patients, respectively, although, did not manage to reach statistical significance, but showed a propensity of patients with MPO-ANCA(+) to impaired renal function. There are several cohorts that showed a higher mortality rate in patients with MPO-ANCA than those with patients with PR3-ANCA, but this difference is usually not statistically significant after adjustment for age, mostly because there seems to be a 10-year discrepancy between the two groups of patients; PR3-ANCA(+) vasculitis is common among 45–55-year-old patients, while MPO-ANCA(+) vasculitis is common in 60–65-year-old patients (13, 23, 24). Overall, both the renal and mortality prognosis seems to be the same for each group of patients and is independent of the ANCA type (5).

With respect to the relapse rate, however, we found that there was a clear dominance in patients with PR3-ANCA(+), with the frequency of patients who had at least one relapse during the 2 years follow-up, reaching to more than four times compared to patients with MPO-ANCA(+) and ANCA(-). Our results were in accord to the literature, as the most important risk factor for relapse of the disease seems to be the presence of PR3-ANCA(+), which is accompanied by a 2-fold higher risk for relapse (5, 25). Interestingly, MPO-ANCA(+) disease has the propensity to relapse later and this could possibly explain the increased differences in relapse rate in this study. The high relapse rate of patients with PR3-ANCA vs. MPO-ANCA does not seem to have an impact on their long-term survival (26). This could be explained by the fact that patients with MPO-ANCA(+) have poorer renal outcomes, worse renal disease, they are more HD dependent, and have more chronic lesions in kidney biopsy than MPO-ANCA(+) vasculitis (27).

In this study, we confirm the distinct clinical phenotypes of MPO-ANCA(+) and PR3-ANCA(+) disease; we describe differences in histology, but, in addition, we point out the similarities between MPO-ANCA(+) and ANCA(-) vasculitis. Response to treatment and outcome of renal function were similar in the three groups, regardless the type of ANCA. PR3-ANCA(+), however, seemed to be more “aggressive,” presenting with a great spectrum of organ involvement and relapsing early and frequently during follow-up. MPO-ANCA(+) disease, on the other hand, is still and quiet and when it is diagnosed, it has usually caused irreversible damage with advanced fibrosing lesions in renal biopsy. ANCA(-) disease acts as a discrete disease, which definitely reminds MPO-ANCA(+) disease. However, although our findings come from a prospective close follow-up of patients, certainly more longitudinal studies are needed to establish the distinction of clinical entities based on the presence of ANCA types, rather than the clinical phenotypes.

## Data Availability Statement

The raw data supporting the conclusions of this article will be made available by the authors, without undue reservation.

## Ethics Statement

The studies involving human participants were reviewed and approved by Ethics Committee of the Hippokration General Hospital, Thessaloniki, Greece, Approval Number 12/16. The patients/participants provided their written informed consent to participate in this study.

## Author Contributions

KB, MS, and AP contributed to conception and design of the study. KB wrote the first draft of the manuscript. MS supervised the work and revised the paper. KB and SK organized the database. CN assessed renal biopsies. GL performed the statistical analysis. ZM and FI wrote sections of the manuscript. AF assessed patients immunological results. All authors contributed to manuscript revision, read, and approved the submitted version.

## Conflict of Interest

The authors declare that the research was conducted in the absence of any commercial or financial relationships that could be construed as a potential conflict of interest.

## Publisher's Note

All claims expressed in this article are solely those of the authors and do not necessarily represent those of their affiliated organizations, or those of the publisher, the editors and the reviewers. Any product that may be evaluated in this article, or claim that may be made by its manufacturer, is not guaranteed or endorsed by the publisher.
